# [Retracted] Platelet-derived growth factor BB mediates the glioma-induced migration of bone marrow-derived mesenchymal stem cells by promoting the expression of vascular cell adhesion molecule-1 through the PI3K, P38 MAPK and NF-κB pathways

**DOI:** 10.3892/or.2025.9037

**Published:** 2025-12-08

**Authors:** Yi Hu, Peng Cheng, Jiang-Chun Ma, Yi-Xue Xue, Yun-Hui Liu

Oncol Rep 30: 2755–2764, 2013; DOI: 10.3892/or.2013.2780

Following the publication of the above paper, it was drawn to the Editor's attention by a concerned reader that areas of the cellular images shown in Fig. 3A and B appeared to be identical to data shown in Fig. 1 of an article published in *Journal of Molecular Neuroscience* a year earlier (in 2012) by the same research group, although in that case, these data were used to represent different experiments. Moreover, comparing the two publications, for the same data, the bar charts showing the ‘number of migrating BMSCs’ reported very different average measurements (~38 in the *Journal of Molecular Neuroscience* paper, and ~220 in the paper above).

Owing to the fact that the abovementioned data had been re-used in the above paper in an unrelated experimental context, the Editor of *Oncology Reports* has decided that this paper should be retracted from the Journal on account of a lack of confidence in the presented data. The authors were asked for an explanation to account for these concerns, but the Editorial Office did not receive a reply. The Editor apologizes to the readership for any inconvenience caused.

